# Classification and Association Analysis of Gerbera (*Gerbera hybrida*) Flower Color Traits

**DOI:** 10.3389/fpls.2021.779288

**Published:** 2022-01-25

**Authors:** Yiwei Zhou, Mao Yin, Farhat Abbas, Yue Sun, Ting Gao, Fulong Yan, Xinyue Li, Yunyi Yu, Yuechong Yue, Rangcai Yu, Yanping Fan

**Affiliations:** ^1^The Research Center for Ornamental Plants, College of Forestry and Landscape Architecture, South China Agricultural University, Guangzhou, China; ^2^Guangdong Key Laboratory for Innovative Development and Utilization of Forest Plant Germplasm, South China Agricultural University, Guangzhou, China; ^3^College of Life Sciences, South China Agricultural University, Guangzhou, China

**Keywords:** Gerbera, flower color, EST-SSR, transcriptome, association analysis

## Abstract

Floral color plays a crucial role in plant life such as plant-pollinator interactions and modifying the abiotic environment of reproductive structures. In the current study, 123 gerbera accessions were divided into six color groups (white, yellow, orange, pink, red, and purple), based on Royal Horticultural Society Color Chart calibration and colorimeter measurement. Partial least squares discriminant analysis showed that the white group was mainly affected by L* value, a* value, *C* value, and total anthocyanin contents, while the yellow group was positively correlated with L* value, b* value, and total anthocyanin contents. Similarly, the orange group was mainly affected by b* value and total carotenoid contents, whereas the pink group was positively correlated with L* and *h* values. Furthermore, the red group was affected by L* value, a* value, *C* value, and total anthocyanin contents, whilst the purple group was mainly distributed by L* value, a* value, b* value, and total anthocyanin contents. Based on ‘Jin Xiang’ transcriptome data, 14,106 expressed sequence tag (EST)-SSR markers were identified and 48 pairs of primers (19 newly developed primers) were screened. Population genetic structure, neighbor-joining clustering, and principal coordinate analysis showed that 123 gerbera accessions could be divided into two groups. EST-SSR-based association analysis showed that 1, 1, 2, 1, 1, 2, and 1 significant loci were related to L*, a*, b*, *C*, and *h*, total carotenoid, and total anthocyanin contents, respectively. These results provide an important reference for flower color classification and genetic improvement of gerbera.

## Introduction

Flowering plants have evolved a wide range of flower kinds exhibiting a striking range of colors using a diversity of numerous pigments (Tanaka et al., 2008; Ng et al., 2018). Petal colors play a decisive role in the survival and successive reproductive process of several ornamental plants via attracting the insects to ensure pollination (Ayala-Silva and Meerow, 2006). Petal colors appeal to flower visitors such as bees and birds which assist the efficient transfer of pollens between conspecific plants (Chittka et al., 1999; Shrestha et al., 2013; Abbas et al., 2017). It can also indicate the presence of pigments and chemicals within a certain species, like anthocyanin, flavonoid pigments, and other essential plant chemicals directly related to the plant’s color (Zhao and Tao, 2015; Abbas et al., 2021; Ke et al., 2021). Furthermore, petal color indicates the expressed genes that provide researchers with a valuable pathway toward generating new varieties and understanding genetic interactions in the breeding program of ornamental plants (Ayala-Silva and Meerow, 2006). The apparent color of the flower is governed due to the choosy absorption of specific wavelengths of light by the petals and via light scattering in the petal’s interior (van der Kooi et al., 2016; Post and Schlautman, 2020). However, the variations in pigments in flowering plants are not well understood.

The quantification of color is a key prerequisite for flower color classification. Recently, Commission Internationale de l’Eclairage (CIELAB) color space, also known as CIE L*a*b*, has been extensively used to quantify the flower and fruit color phenotypes. The CIELAB color space system is defined by using the coordinate axes of L* (lightness), a* (green to red), and b* (blue to yellow). The CIELAB standard provides an accurate and uniform color measurement standard via calculating color on three axes (Hill et al., 1997). This color system has been widely applied in the classification and determination of flower and fruit coloration in several plants including *Manihot esculenta* (Afonso et al., 2017; Moresco et al., 2017), *Paeonia* (Wang et al., 2001), *Chrysanthemum morifolium* (Li et al., 2016), *Osmanthus fragrans* (Wang et al., 2018), *Hemerocallis* spp. (Cui et al., 2019), *Rosa* spp. (Wan et al., 2019), etc. Different flower color is attributed to various pigments that can be divided into three main groups including carotenoids, flavonoids, and betalains (Grotewold, 2006). From phenotype to pigment analysis, understanding the flower color formation of various ornamental plants is critical.

*Gerbera hybrida* (2n = 2x = 50) is one of the most significant ornamental plants that belongs to the family Compositae. *Gerbera hybrida* is highly heterozygous and originated from the crossing of two wild African species (*Gerbera jamesonii* and *Gerbera viridifolia*) (Hansen, 1999). It is valued due to its exceptional and appealing flower colors and forms. Gerbera is a popular greenhouse flower that ranks fifth in the global cut flower market. Moreover, diverse variation in flower color, patterning of ray and disk florets, and abundance of secondary metabolites derived from linked pathways make it a model crop for biosynthetic research (Teeri et al., 2006; Fu et al., 2016). There are few studies available on gerbera flower color, yet there is lack of systematic evaluation and classification of flower color phenotype.

Molecular markers have evolved into an effective tool for cultivar development and breeding programs in a variety of crops (Varshney et al., 2005; Gong and Deng, 2010). Molecular markers are normally used for precise evaluation of genetic relationships and diversity, early selection of genotypes, identification of cultivars, and efficient mapping and tagging of desired genes. Simple sequence repeat or microsatellite (SSR) markers have been extensively used due to their high abundance, multi-allelic nature, co-dominant inheritance, high abundance, hyper-variance, extensive genomic coverage, multi-allelic nature, reproducibility, and co-dominant inheritance (Varshney et al., 2005). SSR markers are normally 1–6 bp long tandem repeats of nucleotide motifs and are considered to be one of the best markers for numerous studies (Schlötterer, 2004). Next-generation sequencing provides an opportunity for batch development of SSRs. Previously, 893 SSR loci were identified from the transcriptome of gerbera and screened 55 markers to analyze the genetic diversity of 40 gerbera accessions (Gong and Deng, 2010, 2012). de Pinho Benemann et al. (2012) screened 17 pairs of primers from 647 pairs of SSR primers that could distinguish 34 gerbera genotypes. In another study, eight microsatellite markers were developed using the magnetic bead enrichment method to analyze the genetic diversity and relationship of 48 gerbera varieties (Chen et al., 2016). Although, there is some progress in the development and application of gerbera SSR markers, yet there is no report on the association analysis of important ornamental traits, which limits the breeding of gerbera.

In the current study, 123 gerbera accessions with different flower colors were used as plant materials. The color variations among all accessions were observed using a colorimeter and RHS Large Color Chart followed by multivariate analysis methods such as hierarchical clustering analysis (HCA) and partial least squares discriminant analysis (PLS-DA). Moreover, total carotenoid and anthocyanin contents were measured from all accessions and new expressed sequence tag (EST)-SSR markers were developed through RNA-seq data. Combining the previous and newly developed EST-SSR markers, the genetic diversity among 123 gerbera accessions was observed. Furthermore, the association analysis of flower color traits was performed first time that will assist in the gerbera breeding and genetic studies.

## Materials and Methods

### Plant Materials

A total of 123 gerbera accessions were collected from different province of China and were grown under identical natural conditions at South China Agricultural University, Guangzhou, China (23.16°N, 113.36°E). The petals of cultivars were collected during the full flowering stage. Three to four biologic replicates of each variety were obtained. For pigment analysis, the collected samples were immediately frozen in liquid nitrogen and stored at –80°C. The detailed information of accessions is listed in Supplementary Table 1.

### Flower Color Measurement and Distinction

At full boom, the colors of petals were described using the RHSCC (Royal Horticultural Society Color Chart) with white background and under the same lighting conditions. Furthermore, a CS-210 precision colorimeter (Hangzhou Caipu Technology, China) was used to quantify the color parameters of the same flowers. The parameters of CIEL*a*b* color coordinate (lightness, L*; chromatic components, a* and b*) were also measured. According to the formula: h=arctan(b*/a*),C=(a+*2b)*21/2, the hue angle *h* and chroma *C* were measured (Gonnet, 1998). As replicates, five measurements were taken for each accession.

### Analysis of Total Carotenoid Content

Total carotenoid (Ct) content was measured as described previously (Singh et al., 2015). Briefly, 2 g fresh flower samples were ground in 10 mL of acetone followed by centrifugation at 5,000 rpm for 8 min. The supernatant was repetitively ground and filtered till devoid of the color. The extract was shifted to a 50 mL flask containing 15 g of anhydrous sodium sulfate. Total carotenoid content was measured using the equation: total carotenoid contents (μ*g*/g) = [(*A*×*V*(ml)× 10^4^)/*A*%cm×*P*(g)]; *A* = absorbance, *V* = total extract volume, *P* = fresh sample weight, *A*% cm = 2,592 (extinction coefficient). The experiment was performed in three to five replicates.

### Analysis of Total Anthocyanin Content

Total anthocyanin (At) content was measured as explained previously with minor modifications (Ai et al., 2016). In short, ∼300 mg of flower petals per sample was collected and ground in liquid nitrogen into a fine powder. To extract anthocyanins, the fine ground powder was transferred to a 5 mL extraction solution comprising hydrochloric acid and methanol mixture (1:99 v/v). Thereafter, the extract solution was incubated at 4°C for 24 h followed by centrifugation at 13,000 rpm for 20 min. Then, the supernatant was shifted to a new tube, and anthocyanin was measured by calculating the optical density (OD) at A_530_ by using a UV-1200 spectrophotometer (MAPADA, Shanghai, China). The measurement of At was conducted using the equation Q_At_ = A_530_ × M^−1^; Q_At_ = amount of At, M = fresh weight (g) of the plant material used for extraction. Data for each sample are reported as the mean of three replicates.

### RNA Extraction, Sequencing, Transcriptome Assembly, and Annotation

Total RNA was isolated using the hexadecyl trimethyl ammonium bromide (CTAB) method with minor modification as described earlier (Tel-Zur et al., 1999; Yue et al., 2014). The quality and quantity of RNA were assessed using the Agilent Bioanalyzer (Agilent Technologies, United States). Furthermore, the integrity of total RNA was evaluated via agarose gel electrophoresis. For RNA-sequencing, the cDNA library was generated using the Illumina Hicseq™ RNA sample prep kit (Illumina, United States) following the manufacturer’s protocols. The size and concentration of the library were evaluated using Qubit 2.0 and Agilent 2100. Through Hi-Seq 2500 sequencing machine, pair-end (150 PE) Illumina high-throughput sequencing was conducted. The transcriptome raw data of gerbera have been deposited in the NCBI database (SRR16889011).

From the raw reads, the low-quality reads and adopters were filtered. The clean reads were *de novo* assembled into contigs with an optimized *k*-mer length = 25 and group pairs distance = 300 using the Trinity program2. The unigenes functions were predicted via BLAST against the NCBI non-redundant protein (Nr), NCBI nucleotide sequences (Nt), and Swiss-Prot databases (*E*-value of 10^–5^). The resulting datasets were validated to the Protein family (Pfam) database with HMMER (E-value 10^–10^). Unigene sequences were aligned against databases of the Gene Ontology (GO) (Ashburner et al., 2000), Kyoto Encyclopedia of Genes and Genomes (KEGG) (Kanehisa et al., 2004), euKaryotic Orthologous Groups (Koonin et al., 2004).

### Microsatellite Sequence Identification and EST-SSR Genotyping

From the transcriptome data, SSR motifs were identified using the microsatellite identification online program (Beier et al., 2017). The identification standards for dinucleotides (di-), trinucleotides (tri-), tetranucleotide (tetra-), pentanucleotide (penta-), and hexanucleotide (hexa-) motifs were set to six, five, five, five, and five repeats, respectively. The SSR primers were designed using the Primer 3 software (Koressaar and Remm, 2007). The designed primer pairs were assessed using *in silico* PCR with unigene sequences as the template. The primers with several amplicons were screened out.

One hundred and thirty-nine EST-SSR markers were used for genotyping 123 gerbera accessions. Genomic DNA was obtained from the fresh and young leaves of each cultivar using the CTAB procedure. DNA quantification was performed via a Nanodrop ND-1000 spectrophotometer (Thermo Scientific, United States). T100™ Thermal Cycler (BIO-RAD, United States) was used for PCR and the conditions were the same as described previously (Zhou et al., 2021). The amplicons were separated using 8% polyacrylamide gel electrophoresis at 300 V in 1.0 × TBE buffer and visualized with silver staining.

### Data Analysis

SPSS 19.0 program (SPSS Inc., Chicago, IL, United States) was used for statistical analysis and Student’s *t*-test. Correlation analysis was based on Spearman correlation coefficient using R 4.0.5. PLS-DA was implemented with the “mixOmics” package of R (4.0.5.). The regression coefficient network was performed using Cytoscape version 3.6.2 (Cline et al., 2007).

The number of alleles (*Na*), number of effective alleles (*Ne*), Shannon’s index (*I*), observed heterozygosity; (*Ho*), expected heterozygosity (*He*), and the polymorphic information content (*PIC*) were calculated using GenAlEx 6.5 software (Peakall and Smouse, 2006) and PowerMarker version 3.25 (Liu and Muse, 2005). Based on the admixture model, population structure analysis was performed using STRUCTURE 2.3.4 (Pritchard et al., 2000) and web-based software STRUCTURE HARVESTER version 0.6.92 (Earl, 2012). CLUMPP v1.1.275 was used to assess the 20 replicates for optimal alignment cluster (Jakobsson and Rosenberg, 2007). To cluster the genotypes, the neighbor-joining (NJ) method was employed based on Nei’s genetic distance in PowerMarker software. Principal coordinate analysis (PCoA) was employed using the “vegan” and “ape” package in R 4.0.5.

Association analysis was performed using the mixed linear model (MLM, Q + K model) in TASSEL4.3.6 (Bradbury et al., 2007). The significance of marker-trait associations was determined at *P* ≤ 0.01 (Jaiswal et al., 2012). To distinguish the true associations, False discovery rate (FDR) was employed (Benjamini and Yekutieli, 2005).

## Results

### Classification and Variation of Flower Color Traits

**FIGURE 1:**
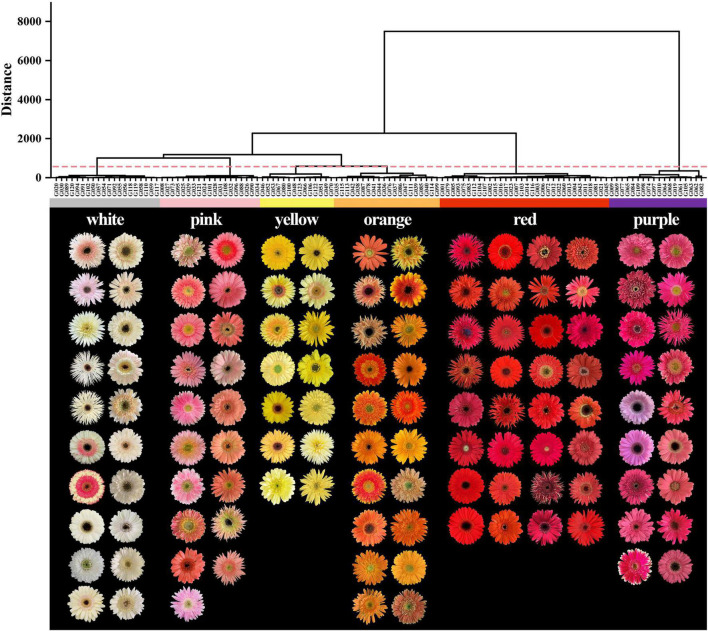
Classification for 123 gerbera accessions based on L*, a*, b*, *C*, and *h* of flower color.

The flower colors of the 123 gerbera accessions ranged from white to purple. The HCA and RHSCC analysis showed that 123 gerbera accessions could be classified into six color groups including white, yellow, orange, pink, red, and purple group (Figure 1 and Supplementary Table 1). Among them, 32 accessions were identified as red group which accounted largest proportion (26.02%) of total gerbera accessions. Similarly, the white and orange groups have 20 gerbera accessions and contributed 16.26% of total gerbera accessions followed by pink (19 accessions, 15.45%), purple (18 accessions, 14.63%), and yellow group (14, 11.38%), respectively (Figure 1).

**FIGURE 2:**
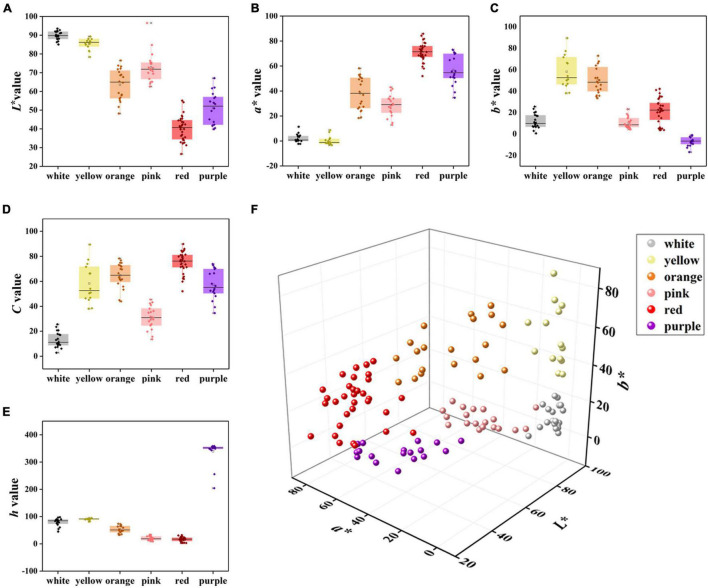
Comparison of L*, a*, b*, *C*, and *h* values of six color groups. **(A)** L* value, **(B)** a* value, **(C)** b* value, **(D)** *C* value, **(E)** *h* value, and **(F)** flower color distribution of 123 accessions based on L*, a*, and b* values.

The L* values of the white and yellow groups were the highest, and the degree of variations among varieties was small, while the L* values of the red and purple groups were the lowest (Figure 2A). The a* value of the white and yellow groups was the smallest and there were negative values in nine accessions, while other four-color groups were distributed in the positive range (Figure 2B). Meanwhile, the red group had the highest a* value with an average of 71.12, followed by the purple, orange, pink, and white group, respectively. The purple group had the smallest b* value, and all of them were less than zero, whereas the b* value of the other color groups was greater than zero (Figure 2C). The b* value of the yellow group was the highest with an average of 58.14, followed by the orange, red, white, and pink groups, respectively. In addition, the *C* value of the red group was highest followed by orange, purple, yellow, pink, and white, respectively. Moreover, the *C* value of the white group was the lowest with an average value of 13.14 (Figure 2D). The *h* value of the purple group was the highest with an average of 358.58, followed by yellow, white, orange, pink, and red, respectively (Figure 2E). The spatial distributions of these six-color groups based on CIEL*a*b* color coordinates were different (Figure 2F) supporting the result of HCA analysis (Figure 1).

### Determination of Total Carotenoid and Anthocyanin

**FIGURE 3:**
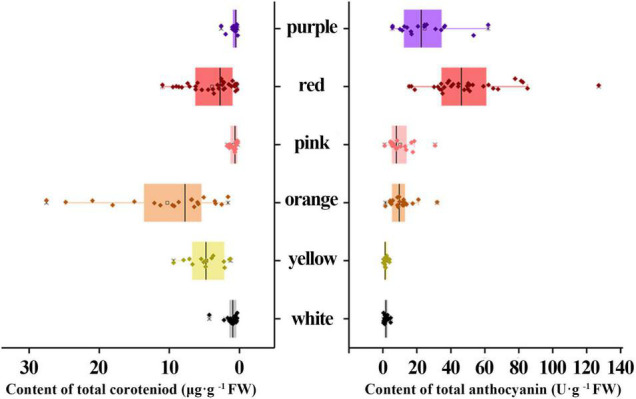
Comparison of the contents of total carotenoid (Ct) and total anthocyanin (At) in different color groups.

The Ct content in the orange group were significantly higher compared to other groups with an average of 10.27 μg g^–1^ FW, followed by yellow (4.72 μg g^–1^ FW) and red groups (3.89 μg g^–1^ FW), respectively (Figure 3). Whereas, Ct contents in white, purple, and pink groups were low. Furthermore, At content in the red group were highest (49.85 U g^–1^ FW), followed by purple (24.54 U g^–1^ FW), orange (10.83 U g^–1^ FW), and pink groups (10.05 U g^–1^ FW). Likewise, At content in the white and yellow groups were the least (Figure 3).

### Correlation and Partial Least Squares Discriminant Analysis

**FIGURE 4:**
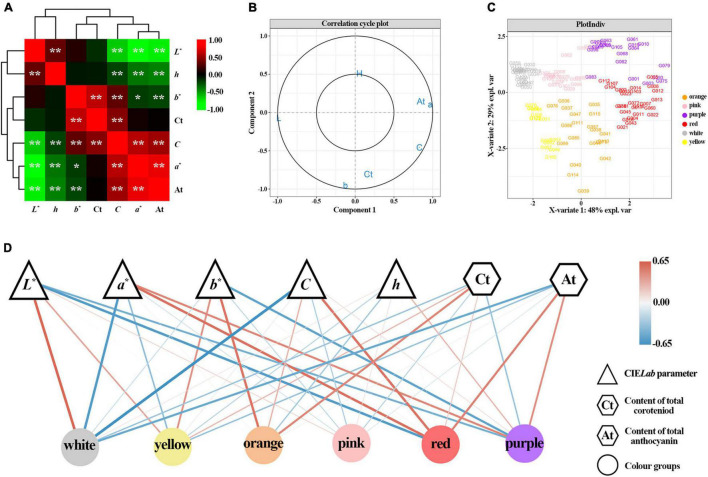
Correlation and partial least squares discriminant analysis (PLS-DA) based on flower color data of 123 accessions. **(A)** Correlation heatmap among L*, a*, b*, *C*, *h*, Ct, and At. *Denotes significant level of 0.05. **Denotes significant level of 0.01. Plot **(B)** shows the loading plot of L*, a*, b*, *C*, *h*, Ct, and At based on PLD-DA analysis. Plot **(C)** depicts the flower color distribution of 123 accessions. Plot **(D)** disclosed the correlation analysis network built on PLS-DA correlation coefficient.

The correlation analysis showed that seven variables had different degrees of correlation, ranging from -0.9624 to 0.9156 (Figure 4A and Supplementary Table 2). L* value negatively correlated with a* value, *C* value and At content. Moreover, a* value negatively correlated with b* and *h* value, while positively correlated with *C* value and At content; b* value positively correlated with *C* value and Ct contents, while negatively correlated with At contents; *C* value negatively correlated with *h* value, while positively correlated with Ct and At contents; *h* value negatively correlated with At content. The analysis showed that b* and *C* value were mainly influenced by Ct content, while L* and a* value were mainly influenced by At content.

To analyze the effects of different chromaticity parameters (L*, a*, b*, *C*, and *h*) and pigments (Ct and At) on different color groups, a PLS-DA analysis was performed. The cut-off graph shows that seven variables are between two confidence ellipses, and the score graph shows that each color group is distinguished, which indicates that the constructed model can well explain the differences of different color groups (Figures 4B,C). From each color group, the white group was mainly affected by L* value, a* value, *C* value, and At content with regression coefficients of 0.61, –0.59, –0.65, and –0.50, respectively (Figure 4D and Supplementary Table 3). The yellow group positively correlated with L* value, b* value, and At content, while negatively correlated with a* value, *h* value, and Ct content, while the absolute value of the regression coefficient of *C* value is low. In the orange group, b* value and total Ct content were the main factors, with regression coefficients of 0.55 and 0.47, respectively. The pink group was positively correlated with the L* value and *h* value, and the regression coefficients of all variables were less than 0.3. The red group was mainly affected by L*, a*, *C*, and At content, with regression coefficients of –0.59, 0.58, 0.57, and 0.50, respectively. Moreover, the red group positively correlated with Ct content. The purple group was mainly affected by L*, a*, b* and At content, with regression coefficients of –0.45, 0.46, –0.55, and 0.43, respectively. Furthermore, the purple group had a negative correlation with Ct content.

### Transcriptome Sequencing, *de novo* Assembly, and Unigenes Annotation

After filtering of the raw sequencing data, 57,133,428 clean reads (∼8.57 Gb) were obtained from the transcriptome. RNA-Seq statistics for the tissue are listed in Supplementary Table 4. More than 94.49% of reads had Q30 or higher quality scores. Using the Trinity *de novo* assembly program, the ∼8.57 Gb high-quality reads were assembled into contigs, transcripts, and unigenes. The statistical data of the assemblies are shown in Supplementary Tables 5, 6. A total of 182,868 transcripts (mean length 486 bp) representing 111,709 unigenes were assembled (mean length 870 bp). The number of transcripts between 200 and 500 bp was the highest, accounting for 51.05%, while the number of genes between 500 and 1,000 bp was the lowest (32.88%).

**FIGURE 5:**
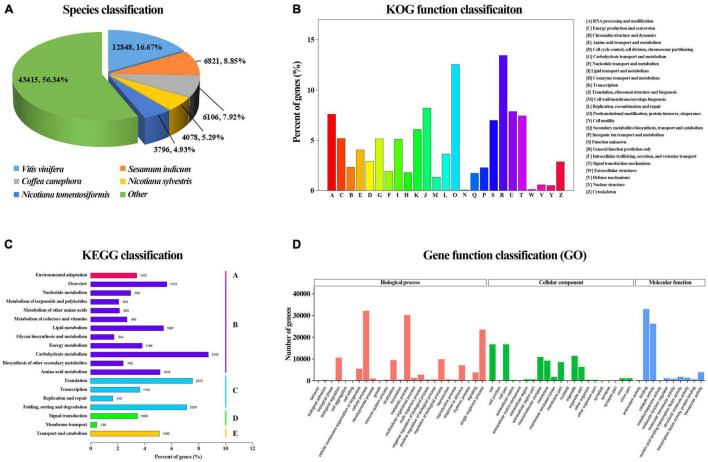
Correlation and PLS-DA analysis based on flower color data of 123 accessions. **(A)** Species classification based on NR database. **(B)** KOG function classification. **(C)** Kyoto Encyclopedia of Genes and Genomes (KEGG) classification. **(D)** Gene function classification.

Searches against the GO, KEGG, KOG, Nr, Nt, and Swiss-Prot databases yielded a total of 111,709 annotated unigenes. 11,295 (10.11%) of them had hits in all seven databases. The unigene hits in each database are shown in Supplementary Table 7 with the number of annotated unigenes being the highest in the Nr (81,057) and the lowest in KOG (21,279). Further BLAST searches against other databases showed that 48,034, 31,484, 60,911, 56,206, and 56,897 unigenes had at least one match in the Nt, KEGG, Swiss-Prot, Pfam, and GO databases, respectively (Supplementary Table 7). In BLASTx homology searches (at cutoff *E*-value of 10^–5^), the five top species with hits in the Nr database were: *Vitis vinifera* (12,848, 16.03%), *Sesamum indicum* (6,821, 8.51%), *Coffea canephora* (6,106; 7.62%), *Nicotiana sylvestris* (4,078, 5.09%) and *Nicotiana tomentosiformis* (3,796, 4.74%) (Figure 5A).

In KOG analysis, 21,279 unigenes were classified into 25 clusters (Figure 5B). Among the 25 KOG categories, the cluster related to general function prediction (2,859 or 13.44%) was the largest, followed by the clusters of posttranslational modification, protein turnover, chaperones (2,671 or 12.55%) and translation, ribosomal structure, and biogenesis (1,743 or 8.19%). The clusters represented by the least number of unigenes were cell motility and extracellular structures (40, 0.19%).

Kyoto Encyclopedia of Genes and Genomes pathway analysis of the 31,484 unigenes revealed that 23,584 (74.91%) could be assigned to 19 pathways belonging to five major categories: metabolism (13,425), genetic information processing (6,261), cellular processes (1,599), environmental information processing (1,227), and organismal systems (1,072) (Figure 5C). The category with the largest number of unigenes was metabolism, in which the most represented four pathways that were carbon metabolism (ko01200) (987, 7.35%), starch and sucrose metabolism (ko00500) (888, 6.61%), biosynthesis of amino acids (ko01230) (832, 6.20%), and purine metabolism (ko00230) (761, 5.67%).

In addition, 56,897 unigenes that matched the GO database were classified into 56 functional sub-groups of the three main GO groups: biological process, cellular component and molecular function (Figure 5D). The majority of the unigenes were assigned to biological processes (140,724, 47.56%), followed by cellular components (86,093, 29.09%), and molecular functions (69,097, 23.35%). Under the category of biological processes, cellular processes (32,193, 22.88%) and metabolic processes (30,201, 21.46%) were predominant. In the cellular component category, cell part (16,753, 19.46%) and cell (16,749, 19.45%) were the most abundant classes. As for the molecular function, catalytic activities (32,996, 47.75%) and binding (26,246, 37.98%) were the top two categories in numbers.

### Identification and Distribution of Simple Sequence Repeats in ‘Jin Xiang’ Transcriptome

A total number of 14,106 SSR loci with motif types were discovered. Di- repeat motifs (8,452, 59.92%) were predominated among the total SSRs identified, followed by Tri- repeat motifs (5,333, 37.81%), Tetra repeat motifs (138, 0.98%), Hexa- repeat motifs (128, 0.91%), and Penta- repeat motifs (55, 0.39%). For the Di- and Tri- repeat motifs, the frequency was as high as 97.73% in total. The number of motifs types for Di-, Tri-, Tetra-, Penta-, and Hexa- were 8, 30, 34, 18, and 67, respectively (Supplementary Table 8).

**FIGURE 6:**
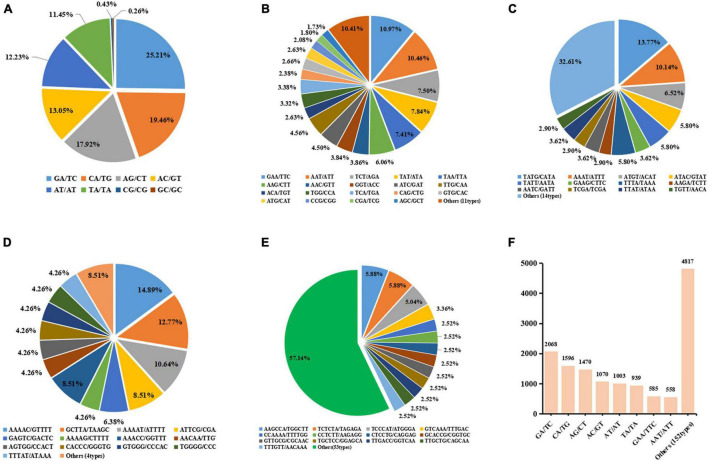
Proportion of different repeat motif types in Dinucleotides **(A)**, Trinucleotides **(B)**, Tetranucleotide **(C)**, Pentanucleotide **(D)**, and Hexanucleotide **(E)**. Plot **(F)** shows the most copious motif types with number of >500.

The number of SSR loci gradually decreased as the number of repeat times increased (Table 1). Among them, SSR loci with five repetitions were the foremost. The most abundant Di- motif was GA/TC, responsible for 25.21% of Di- motif repeats followed by CA/TG (19.46%). In Tri- repeats, GAA/TTC (10.97%) was the most abundant, followed by AAT/AAT accounting for 10.46%. In Tetra- repeats, TATG/CATA (13.77%) has the largest number and AAAT/ATTT (10.14%) was the second, while the other motif accounts for less than 7%. In Penta- repeats, AATCG/CGATT (14.55%) has the largest proportion, followed by AAAAC/GTTTT (12.73%) and GCTTA/TAAGC (10.91%). In Hexa- repeats, the most abundant motif types are AACCAA/TTGGTT, accounting for 7.03%, while the number of other motifs types was less than 30 (Figures 6A–E and Supplementary Table 9). The five types of motifs with a number of more than 1,000 are shown in Figure 6. The most copious motif (>500) was GA/TC (2,068, 14.66%) followed by GA/TG (1,596, 11.31%), AG/CT (1,470, 10.42%), AC/GT (1,070, 7.59%), AT/AT (1,003, 7.11%), TA/TA (939, 6.66%), GAA/TTC (585, 4.15%) and AAT/ATT (558, 3.96%), respectively. The total number of the remaining 149 motif types was 4,567 only (Figure 6F and Supplementary Table 9).

**TABLE 1:** Frequencies of different simple sequence repeat (SSR) repeat motif types.

Number of repeats	Di-	Tri-	Tetra-	Penta-	Hexa-	Total	Percentage (%)
5	0	2,998	101	50	92	3,241	22.98
6	1,820	1,288	29	1	24	3,162	22.42
7	1,227	507	2	3	4	1,743	12.36
8	1,013	299	1	0	6	1,319	9.35
9	665	62	3	0	1	731	5.18
10	445	56	0	0	1	502	3.56
11–20	2,556	121	2	1	0	2,680	19.00
>20	726	2	0	0	0	728	5.16
Total	8,452	5,333	138	55	128	14,106	100
Percentage (%)	59.92	37.81	0.98	0.39	0.91	100	

### Genetic Diversity Analysis Based on EST-SSR Markers

To develop polymorphic SSR markers suitable for 123 accessions, 139 pairs of SSR primers were selected, including 53 previously developed EST-SSR primers and 86 newly developed EST-SSR primers. We used a PAGE analysis to screen out 48 pairs of SSR primers with a good amplification effect and polymorphism to analyze 123 gerbera accessions. Among 48 SSR loci, 3 are Di-, 28 are Tri-, 4 are Tetra-, 3 are Penta-, 8 are Hexa-, and 2 are complex repetition types with lengths ranging from 12 to 54 bp (Supplementary Table 10). The 48 pairs of primers can amplify 155 alleles in the test accessions (Table 2). The *PIC* values ranged from 0.20 to 0.77 with an average of 0.44, indicating that these plants showed middle genetic diversity. The number of observed alleles (*Na*) ranged from 2 to 7 with an average allele number of 3.21. The number of effective alleles (*Ne*) ranged from 1.26 to 5.03 with an average allele number of 2.25. The Shannon information index (*I*) ranged from 0.38 to 1.76 with an average of 0.86. The observed heterozygosity (*Ho*) ranged from 0.03 to 0.81 with an average of 0.45. The expected heterozygosity (*He*) ranged from 0.21 to 0.80 with an average of 0.50. Among 48 SSR primers, GEM65 yielded the highest number of alleles (7 alleles), with a *PIC* of 0.77.

**TABLE 2:** Characteristics of 22 polymorphic SSR loci in 123 gerbera accessions.

Primer	SSR motif	*Na*	*Ne*	*I*	*Ho*	*He*	*PIC*	Origin
P2-15	(AG)23	3	2.67	1.04	0.27	0.63	0.55	Newly developed
P3-3	(ATA)14	5	4.25	1.52	0.76	0.76	0.73	Newly developed
P3-4	(GAT)11	5	4.40	1.54	0.50	0.77	0.74	Newly developed
P3-18	(GTT)7	2	1.88	0.66	0.43	0.47	0.36	Newly developed
P3-19	(GCG)7	4	3.29	1.27	0.74	0.7	0.64	Newly developed
P3-24	(CGG)6	3	1.80	0.78	0.49	0.44	0.40	Newly developed
P3-28	(GCC)9	3	1.49	0.59	0.16	0.33	0.29	Newly developed
P4-1	(ATGT)8	4	1.26	0.47	0.21	0.21	0.20	Newly developed
P4-5	(AATC)6	2	1.63	0.57	0.44	0.39	0.31	Newly developed
P5-2	(AAACC)5	3	2.05	0.77	0.58	0.51	0.40	Newly developed
P5-4	(GCTTA)5	3	2.48	0.97	0.54	0.60	0.51	Newly developed
P6-1	(CGACAA)8	3	1.68	0.72	0.32	0.41	0.36	Newly developed
P6-3	(GAACCA)7	4	1.84	0.71	0.54	0.46	0.37	Newly developed
P6-5	(ACAGAA)6	3	1.37	0.49	0.32	0.27	0.24	Newly developed
P6-7	(AACCAA)5	4	1.67	0.73	0.46	0.4	0.36	Newly developed
P6-8	(ATGTGT)5	3	1.49	0.58	0.24	0.33	0.29	Newly developed
P6-9	(GTCAAG)5	4	1.77	0.69	0.52	0.44	0.35	Newly developed
P6-11	(AGAAC)5	2	1.7	0.6	0.10	0.41	0.33	Newly developed
PC-2	(A)11(AC) 9*ataa caagagt(A)13	2	1.74	0.62	0.46	0.42	0.33	Newly developed
GEM8	(AAACC)6	2	1.54	0.54	0.33	0.35	0.29	Gong and Deng, 2012
GEM14	(ACC)6	2	2.00	0.69	0.03	0.50	0.38	Gong and Deng, 2012
GEM44	(CATA)8	4	2.46	1.11	0.43	0.59	0.55	Gong and Deng, 2012
GEM57	(CGG)6	3	2.03	0.80	0.55	0.51	0.41	Gong and Deng, 2012
GEM63	(TTA)6	2	1.91	0.67	0.50	0.48	0.36	Gong and Deng, 2012
GEM65	(CT)16(CA)11	7	5.03	1.76	0.73	0.80	0.77	Gong and Deng, 2012
GEM78	(AAT)6	2	1.29	0.38	0.20	0.22	0.20	Gong and Deng, 2012
GEM109	(TCT)8	3	2.04	0.84	0.52	0.51	0.43	Gong and Deng, 2012
GEM126	(TTC)7	3	1.90	0.83	0.49	0.47	0.42	Gong and Deng, 2012
GEM130	(TCGA)5	2	1.43	0.48	0.33	0.30	0.25	Gong and Deng, 2012
GEM132	(CTT)7	4	2.61	1.16	0.61	0.62	0.57	Gong and Deng, 2012
GEM133	(TG)14	3	2.38	0.98	0.50	0.58	0.51	Gong and Deng, 2012
GEM140	(GTA)6	4	2.14	1.02	0.59	0.53	0.49	Gong and Deng, 2012
GEM148	(CAG)6	5	3.83	1.48	0.6	0.74	0.7	Gong and Deng, 2012
GEM162	(AAT)11	4	2.58	1.06	0.45	0.61	0.54	Gong and Deng, 2012
GEM163	(TAA)7	3	2.05	0.88	0.49	0.51	0.45	Gong and Deng, 2012
GEM187	(TCT)6	5	4.39	1.54	0.81	0.77	0.73	Gong and Deng, 2012
GEM201	(ATA)6	5	3.84	1.46	0.59	0.74	0.70	Gong and Deng, 2012
GEM203	(AAT)9	3	2.13	0.89	0.48	0.53	0.46	Gong and Deng, 2012
GEM209	(TTA)8	2	1.85	0.65	0.31	0.46	0.35	Gong and Deng, 2012
GEM213	(ACC)6	2	1.41	0.46	0.28	0.29	0.25	Gong and Deng, 2012
GEM225	(ACC)5	2	1.41	0.46	0.35	0.29	0.25	Gong and Deng, 2012
GEM226	(GTG)5	2	1.66	0.59	0.40	0.40	0.32	Gong and Deng, 2012
GEM233	(TAT)5	2	1.70	0.60	0.41	0.41	0.33	Gong and Deng, 2012
GEM234	(TAT)5	3	1.98	0.86	0.53	0.49	0.44	Gong and Deng, 2012
GEM261	(CAAAG)4	4	3.24	1.28	0.73	0.69	0.64	Gong and Deng, 2012
GEM264	(AAT)8	2	1.83	0.65	0.42	0.45	0.35	Gong and Deng, 2012
GEM289	(AT)6	3	2.92	1.09	0.66	0.66	0.58	Gong and Deng, 2012
GEM294	(GAA)7	4	1.75	0.78	0.46	0.43	0.38	Gong and Deng, 2012
Mean		3.22	2.18	0.82	0.42	0.48	0.44	

*Na, number of allele; Ne, number of effective allele; I, Shannon’s index; Ho, observed heterozygosity; He, expected heterozygosity; PIC, polymorphic information content.*

**FIGURE 7:**
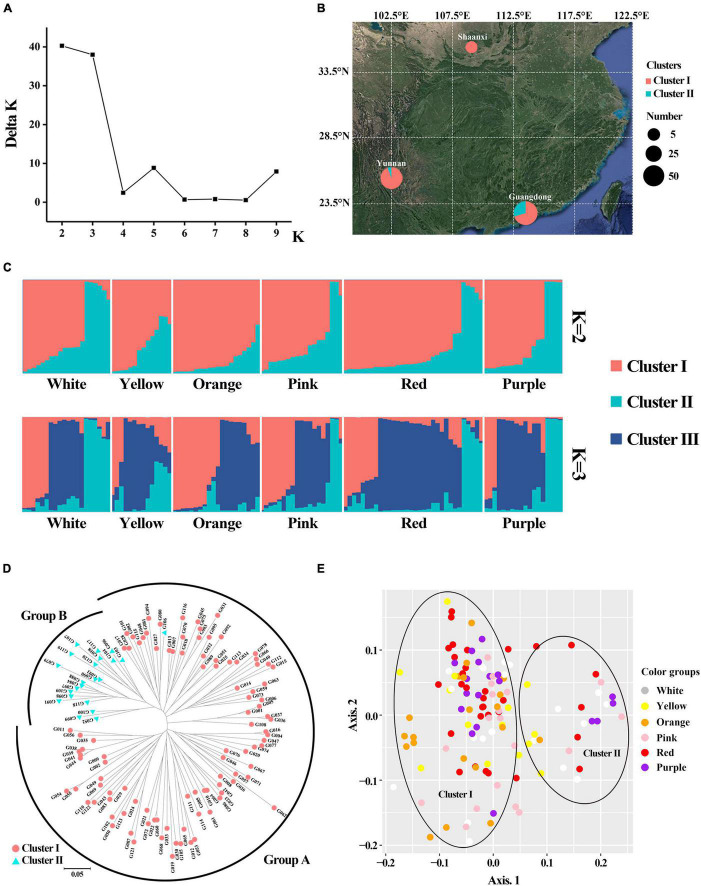
Genetic structure and cluster analysis for 123 gerbera accessions based on 48 EST-SSR. **(A)** The value of Δ*K* and *K* in populations structure analysis. **(B)** Grouping of populations according to structure (*K* = 2) and their geographical source. **(C)** Population structure analysis (*K* = 2 and 3). **(D)** Neighbor-joining tree. **(E)** Principal coordinate analysis.

Population structure analysis (PSA) revealed that when *K* = 2, ΔK was the largest, and 123 accessions were divided into two clusters (Figures 7A,C) and ΔK was the second largest when *K* = 3. NJ cluster analysis and principal coordinate analysis (PCoA) also revealed that there were two major clusters among the gerbera population (Figures 7D,E). For each *Q*-value, genotypes with membership probability >60% were assigned to the same group, while those with <60% probability in any group were assigned as “admixed” (Lu et al., 2009). When *K* = 2, cluster I include 101 accessions with an average *Q* value of 0.8516 (72.28% of them with *Q* > 0.80). Cluster II contains 22 accessions with an average *Q* value of 0.8659 (72.73% of them with *Q* > 0.80). The *Q* value of six accessions was less than 0.6, indicating that they were admixed in which four accessions (G006, G081, G108, and G122) belong to cluster I and 2 accessions (G052 and G099) belong to cluster II. There was no significant difference in petal color group among different clusters for flower color characters (Figures 7C,E and Supplementary Figure 1). However, in cluster I, the flower core color of 58 accessions were dark, while 45 accessions were light (Supplementary Table 1). In cluster II, 86.36% of accessions’ flower core were dark-colored, and only three accessions’ flower core (G099, G106, and G119) were light-colored (Supplementary Table 1). In terms of geographical origin, cluster I include 50 accessions from Yunnan, six accessions from Shaanxi, and 47 accessions from Guangdong province (Figure 7B and Supplementary Table 1). In cluster II, accessions from Guangdong province accounted for 86.36% and only three accessions were collected from Yunnan (Figure 7B and Supplementary Table 1).

Neighbor-joining cluster analysis was consistent with the population structure analysis (Figure 7D). The analysis showed that 123 accessions in the NJ tree were divided into two groups. Group A includes all accessions in cluster I and one admixed accession (G106) in cluster II, while Group B includes 21 accessions in cluster II. The two clusters were also supported by PCoA with 48 EST-SSR markers (Figure 7E). The boundary between the two clusters was obvious. The total proportions of the variation explained by the first and second principal components were 6.48 and 4.74%, respectively.

### Association Analysis of Flower Color-Related Traits

**FIGURE 8:**
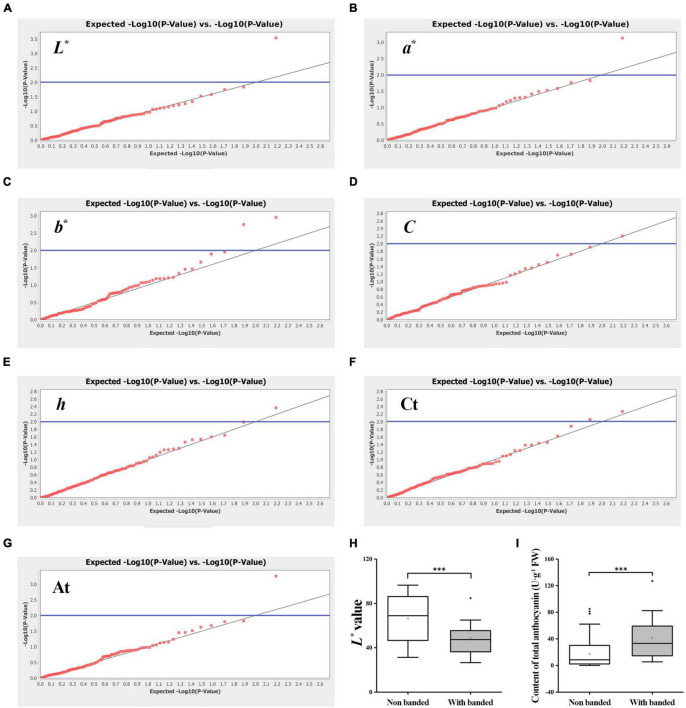
Association analysis of EST-SSR markers with seven flower colors related traits among 123 gerbera accessions. **(A)** QQ plot of L* value. **(B)** QQ plot of a* value. **(C)** QQ plot of b* value. **(D)** QQ plot of *C* value. **(E)** QQ plot of *h* value. **(F)** QQ plot of total carotenoid content. **(G)** QQ plot of total anthocyanin content. Threshold: the blue line is *P* = 0.01. **(H,I)** Box chart analysis with L* value and total anthocyanin content of GEM65c locus. ^***^Denotes significant level of 0.001.

An association analysis was performed to further obtain the color-related molecular markers of gerbera and to validate the application potential of 48 SSR markers. A mixed linear model (MLM, Q **+** K model) was used to analyze the correlation of Ct and At contents. The findings are shown in Table 3 and Figure 8. The number of markers significantly associated with L*, a*, b*, *C*, and *h* values were 1, 1, 2, 1, and 1, respectively with an average *R*^2^ of 0.08673. Two markers were significantly correlated with Ct content with an average *R*^2^ of 0.06206, while one marker was significantly correlated with At contents. Interestingly, some markers can be associated with multiple traits. GEM65c was significantly associated with L*, a*, *C*, and At, while Gem148c and P3-28b were significantly associated with b* value and Ct, respectively. When using a higher threshold standard (FDR = 0.1), only GEM65c was significantly associated with L* (*R*^2^ = 0.1143) and At (*R*^2^ = 0.1034). It can be seen from Figure 8F that the L* value of the non-banded group was significantly higher than that of a banded group, and Figure 8H shows that the At content of the non-banded group was significantly lower than that of a banded group.

**TABLE 3:** Nine loci with significant association with seven flower color traits in 123 gerbera accessions.

Traits	Locus	Primer	*P*-value	FDR-adjusted *P*-value	*R*^2^ (%)
L*	GEM65c	GEM65	0.0003	0.0448	11.43
a*	GEM65c	GEM65	0.0007	0.1154	9.82
b*	GEM148c	GEM148	0.0011	0.1721	9.15
b*	P3-28b	P3-28	0.0018	0.1387	8.36
*C*	GEM65c	GEM65	0.0063	0.9781	6.33
*h*	GEM203a	GEM203	0.0043	0.6665	6.94
Ct	GEM148c	GEM148	0.0054	0.8324	6.59
Ct	P3-28b	P3-28	0.0087	0.6774	5.82
At	GEM65c	GEM65	0.0005	0.0846	10.34

## Discussion

Flower color is one of the most important characteristics of ornamental plants, and identifying flower color efficiently and accurately is the first step in the genetic improvement of ornamental plants. Based on colorimeter measurements and RHSCC, 811 chrysanthemum resources were divided into eight color groups: white, yellow, purple, light yellow, dark, red, orange, and brown (Hong et al., 2012). In *Osmanthus fragrans*, 24 germplasm resources could be divided into 4 groups by colorimeter data and cluster analysis (Wang et al., 2018). Colorimetry and HCA analysis were used in this study to classify flower colors. Based on HCA analysis of CIELAB parameters (L*, a*, b*, *C*, and *h*) and identification by RHSCC, 123 gerbera accessions were divided into six color groups, which provided a reference for the rapid identification of color characters of gerbera. In the future, colorimeter measurements will be able to accurately and quickly determine the phenotype of gerbera flower color, allowing for selective breeding while avoiding the error caused by human visual perception.

Previous research has shown that carotenoids primarily affect colors ranging from yellow to red, whereas anthocyanins primarily affect colors ranging from orange to purple. Gerbera’s color variation is caused by both carotenoids and anthocyanins (Booy, 1995; Yuan et al., 2013). In some gerbera varieties, total anthocyanin was found to be a key factor affecting the change of color vary from yellow to orange (Kishimoto et al., 2007). Chen et al. (2010) analyzed flower color of 10 gerbera varieties through microscopic observation and colorimetric analysis. They discovered that anthocyanin is the primary pigment in red, purple, and orange-red flowers, that the ratio of anthocyanin to carotenoid determines the final color of orange-yellow flowers, and that carotenoid is the primary pigment in yellow flowers. PLS-DA analysis revealed that total Ct and total At were the most important factors influencing gerbera color group differences in this study which are consistent with the previous findings. Furthermore, the contraption of different pigments to different gerbera color groups were quantified by PLS-DA analysis based on 123 gerbera accessions flower color. The Ct content was the main factor affecting the orange group, while At content was the main factor affecting the red and purple color group of gerbera. Previous studies mainly focused on the biosynthesis and the regulation of anthocyanins (Seitz et al., 2006; Bashandy et al., 2015; Naing et al., 2018; Zhong et al., 2020), however, carotenoids also play an important role in the formation of gerbera color, which should be paid more attention to in the future studies. These results will provide insights into gerbera color characteristics, which will assist to screen breeding materials with excellent flower color characteristics.

SSR molecular marker are considered to be one of the most effective molecular markers. Due to the lack of genomic data, molecular markers of gerbera mainly rely on EST-SSR markers. In this study, 19 pairs of SSR primers were developed based on transcriptome data. These primers can be used to analyze the genetic diversity and construct a genetic map of gerbera. When the *PIC* value is greater than 0.25 and less than 0.5, the level of polymorphism is middle (Botstein et al., 1980). In this study, the average *PIC* value was 0.44, implying the middle genetic diversity of 123 accessions based on 48 EST-SSR markers. Likewise, 53 SSR markers were used to analyze the genetic diversity of 40 gerbera germplasm and the average *PIC* values was 0.426 (Gong and Deng, 2010). However, variation was observed in another study, where they use 17 SSR markers to analyze 34 germplasm with an average value of 0.67 (de Pinho Benemann et al., 2012). These differences may be due to the differences in the number and types of germplasm resources and marker primers. PSA, cluster and principal coordinate analysis showed that 123 gerbera accessions could be divided into two clusters, which could be classified according to the color of the flower center to a certain extent, which are in lined with the previous findings performed in *O*. *fragrans* (Chen et al., 2021). Furthermore, member of gerbera plants from cluster II were mainly belongs to Guangdong province (Figure 7B and Supplementary Table 1). Considering that the gerbera breeding work of Yunnan Province is the earliest in China, the 3 accessions from Yunnan might be the ancestors of other accessions in cluster II. These findings suggest that petal color should not be considered as a single factor in gerbera breeding parent selection, but rather in conjunction with genetic relationship and regional source.

Until now, there is a lack of stable transgenic system in gerbera, so molecular marker-assisted selection (MAS) is still an important means of gerbera color breeding. Although many studies on the color of gerbera flowers have been reported, yet color-related molecular markers have received little attention thus far which restricts the breeding of gerbera flower color. Association analysis has been applied to many plant color traits, and some effective molecular markers have been successfully obtained. Genome-wide association analysis of flower and fruit color of 191 eggplant germplasm resources revealed that some loci are consistent with the reported QTLs (Cericola et al., 2014). Likewise, genome-wide association analysis of *Prunus mume* showed that *MYB108* was associated with the bud, stigma, calyx and petal color of the flowers (Zhang et al., 2018). These results indicate that association analysis with color phenotype is an effective way to find out some potential loci of plant. Additionally, multiple significant loci can be detected in association analysis of complex quantitative traits, but the phenotypic interpretation rate (*R*^2^) is usually low especially in outcrossing plants (Yan et al., 2011; Sun et al., 2015). In this study, the MLM model was used to correlate the flower color-related traits to nine loci, and the *R*^2^ ranged from 5.82 to 11.43%, which also indicates that these quantitative traits were controlled by micro effect polygenes. We found that one locus was significantly associated with two traits at the same time and the correlation analysis revealed the characteristic of one cause and multiple effects. For example, L* value and At content have a strong negative correlation, and both of them were associated with GEM65c. Gerberas typically take more than half a year to bloom after seeding. These important molecular markers can aid in the identification of the flower color phenotype of gerbera at the seedling stage, providing a reference for MAS. For instance, the significant locus GEM65c result shows that in the non-banded group, the flower color of most individuals are light with higher L* value and lower At content. On the contrary, when there is a band, the flower color of most individuals are dark with lower L* value and higher At content. In other words, by using the GEM65 primer for PCR detection of gerbera seedling DNA, the color depth of the seedling’s flower could be better identified, providing important guidance for directional screening of individuals with dark or light flower color.

## Conclusion

In conclusion, 123 accessions were distinctly divided into six color groups based on the colorimeter and RHSCC calibration. The PLS-DA analysis revealed that different color groups were affected by various concentration of L*, a*, b*, *C*, *h*, total carotenoid, and total anthocyanin contents. Furthermore, we performed transcriptome analysis of ‘Jin Xiang’ accession. 14,106 EST-SSR markers were developed from transcriptome data, and 48 pairs of primers, including 19 newly developed primers, were tested. We performed multivariant analysis such as NJ clustering, population genetic structure and PCoA analysis that clustered 123 accessions into two groups. Finally, the association analysis revealed that 6 molecular markers were significantly associated with color-related traits.

## Data Availability Statement

The original contributions presented in the study are publicly available. This data can be found here: https://www.ncbi.nlm.nih.gov/sra/SRR16889011.

## Author Contributions

YZ, MY, and YF conceived and designed the concept. YZ, MY, FA, YS, and TG performed the experiments. FY, XL, YNY, and YHY helped in analysis the data. YZ, FA, YF, and RY drafted and reviewed the manuscript. All authors endorsed the final version of the manuscript.

## Conflict of Interest

The authors declare that the research was conducted in the absence of any commercial or financial relationships that could be construed as a potential conflict of interest.

## Publisher’s Note

All claims expressed in this article are solely those of the authors and do not necessarily represent those of their affiliated organizations, or those of the publisher, the editors and the reviewers. Any product that may be evaluated in this article, or claim that may be made by its manufacturer, is not guaranteed or endorsed by the publisher.

## Supplementary Material

Supplementary Figure 1 Principal coordinate analysis (PCoA) (Axis 3 vs Axis 4) of different flower color groups.

## Abbreviations

SSR: simple sequence repeat
PAGE: polyacrylamide gel electrophoresis
PIC: polymorphism information content
PCR: polymerase chain reaction
QTL: quantitative trait loci
CTAB: cetyl trimethyl ammonium bromide
NJ: neighbor-joining
BP: base pairs
PCoA: principal coordinate analysis.
