# Supplementary figures and images for: Classification and Association Analysis of Gerbera (Gerbera hybrida) Flower Color Traits
Source: Front Plant Sci. 2022 Jan 25;12:779288. doi: 10.3389/fpls.2021.779288 (PMC8824200; doi:10.3389/fpls.2021.779288)

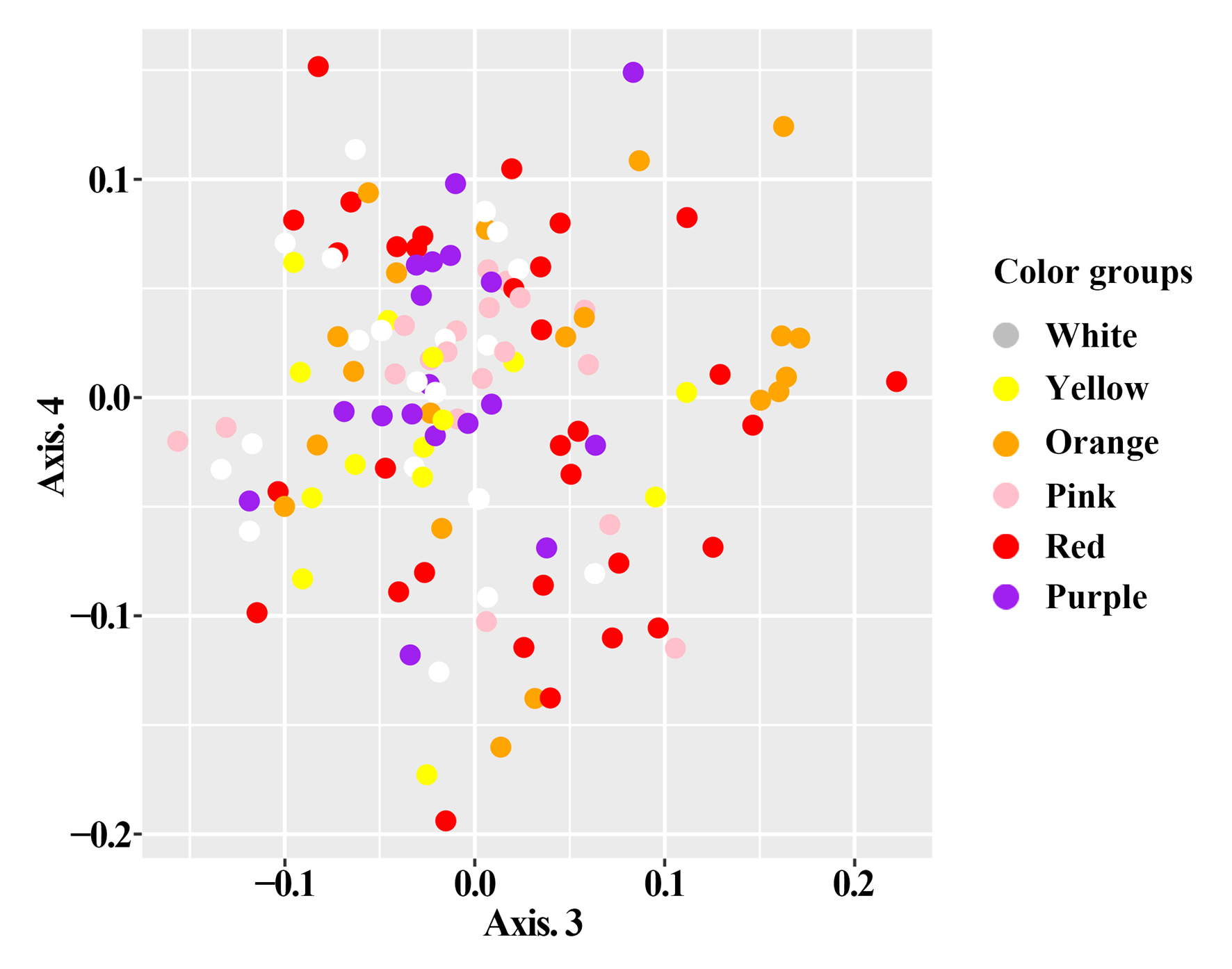

Supplement: Supplementary Figure 1 — Principal coordinate analysis (PCoA) (Axis 3 vs Axis 4) of different flower color groups. [file Image_1.JPEG]
